# Diet–Cognition Associations Differ in Mild Cognitive Impairment Subtypes

**DOI:** 10.3390/nu13041341

**Published:** 2021-04-17

**Authors:** Qiumin Huang, Xiaofang Jia, Jiguo Zhang, Feifei Huang, Huijun Wang, Bing Zhang, Liusen Wang, Hongru Jiang, Zhihong Wang

**Affiliations:** National Institute for Nutrition and Health, Chinese Center for Disease Control and Prevention, 29 Nanwei Road, Beijing 100050, China; qmhuangx@163.com (Q.H.); jiaxf@ninh.chinacdc.cn (X.J.); zhangjg@ninh.chinacdc.cn (J.Z.); fayekobe@163.com (F.H.); wanghj@ninh.chinacdc.cn (H.W.); zzhangb327@aliyun.com (B.Z.); wangls@ninh.chinacdc.cn (L.W.); jianghr@ninh.chinacdc.cn (H.J.)

**Keywords:** diet, food consumption, cognition, cognitive domains, MCI subtypes, China

## Abstract

Cognitive function is not generally associated with diet, and there is debate over that association. Moreover, little is known about such associations with the specific cognitive domains and subtypes of mild cognitive impairment (MCI). We analyzed data of 4309 Chinese adults aged 55 and over from the Community-based Cohort Study on Nervous System Diseases from 2018–2019. Dietary habits were assessed at inclusion using a validated semi-quantitative food frequency questionnaire. Cognitive function of the participants was measured by using the Montreal Cognitive Assessment. Analyses were performed using multiple logistic regression and quantile regression with adjustment for socio-demographic, lifestyle, and health-related factors. Compared with normal cognition participants, those with a worse cognition state were characterized as being an older age and lower economic level. After adjustment for potential factors, participants with higher consumption of rice, legumes, fresh vegetables, fresh fruit, pork, poultry, fish, and nuts tended to have higher scores of global cognitive function and domains, and to have lower odds of MCI, while those with higher consumption levels of wheat and eggs had worse cognition, compared with the corresponding bottom consumption level of each food. Participants with a medium consumption level of beef or mutton had 57% (OR: 1.57, 95%CI: 1.07–2.32) higher odds of aMCI-SD, whereas they had 50% (OR: 0.50, 95%CI: 0.34–0.73) lower odds of naMCI-MD. Similarly, the highest consumption level of dairy was positively associated with the odds of aMCI-SD (OR:1.51, 95%CI:1.00–2.29), but inversely linked to the odds of naMCI-SD (OR: 0.60, 95%CI: 0.38–0.93) and naMCI-MD (OR: 0.49, 95%CI: 0.29–0.82). Most diet global cognitive benefits were observed to be associated with the preexisting higher consumption of rice, legumes, fresh vegetables, fresh fruit, meat, and nuts. In addition, the heterogeneity of associations between the consumption of certain foods and MCI subtypes was observed among Chinese adults aged over 55 years. These cross-sectional observations require validation in prospective studies.

## 1. Introduction

Age-related diseases have gradually imposed a heavy burden on public health worldwide, of which dementia is a primary concern, particularly Alzheimer’s disease (AD), with increases in the proportion of the aging population in both developed and developing countries [1]. In 2020, an estimated 5.8 million Americans age 65 and older were living with AD, and 9.8 million of the Chinese population aged 60 years or older are reported to have AD [2]. Mild cognitive impairment (MCI), an intermediate state between normal cognitive aging and AD, is a known early manifestation of AD and the annual conversion rate from MCI to AD may be 8.1% in a community setting [3], characterized as amnestic or non-amnestic deficit, therefore providing valuable information about the population at risk for developing AD [4]. To date, no effective cure is available to delay the progression of AD [5], while nutrition plays an important role in the aging process of the brain [6]. Hence, it is critical to explore the evidence on the association between dietary factors and MCI risk in the elderly population to shift focus towards prevention methods of this pre-dementia phase of AD.

The links between diets and cognition have been of public interest. Special attention has been devoted to fresh vegetables and fruit because they are a good source of antioxidant nutrients, such as vitamin C, vitamin E, and carotenoids, as well as the consumption of fish and nuts due to their richness in unsaturated fatty acids that were proved to have anti-inflammatory benefits [7,8]. The effect of the consumption of these vegetables and fruits on cognition function have been summarized in a systematic review of nine cohort studies, indicating that high consumption of vegetables was associated with slower rates of cognitive decline in older age, but not fruit consumption [9]. Yet, there are abundant studies on analytically combined fruit and vegetable consumption showing that fruit and vegetable intakes [10,11,12] and, in addition, berry intake [13], have been associated with better cognitive outcomes. Moreover, intervention studies have also shown positive findings in relation to fruit and cognition. For example, in studies in which grape, blueberry, orange, or cherry juice was consumed daily by participants for a period of 8–16 weeks, positive findings in respect to cognition were reported [14]. The increased consumption of fish or nuts was observed to reduce the risk of MCI [7,15,16], benefiting cognitive abilities. Few studies have examined the effect of staple foods [17] or meat [18] that are generally considered to have negative effects on health because of their high contents of carbohydrate or saturated fat. Most previous studies, however, tended to focus on the outcome of MCI, without data specifically on subtypes of MCI, including single domain/multidomain amnestic and non-amnestic subtypes [19], which feature as deficits of distinct cognitive domains related to unequal brain function and have varied prevalence. Moreover, some Chinese studies suggested the above foods may have a beneficial effect on cognitive abilities [20,21,22], but were mainly based on a limited number of respondents recruited in a localized region and there were inconsistent results [21,23]. Therefore, the present study aims to examine the association of diet, covering various foods, with the odds of MCI and its subtypes using a sample of Chinese people aged 55 years and older from the Community-based Cohort Study on Nervous System Diseases (CCSNSD), as well as with a focus on various cognitive domains.

## 2. Materials and Methods

### 2.1. Study Population

Data in the present study were derived from the baseline of the CCSNSD, an ongoing and longitudinal study established from 2018–2019 by the National Institute for Nutrition and Health, Chinese Center for Disease Control and Prevention, which focused on the potential factors associated with risks of three nervous diseases, including epilepsy for subjects aged >1 year, and AD and Parkinson’s disease for the population aged ≥55 years old. Participants without such diseases were enrolled using a multistage stratified random sampling approach in Hebei, Zhejiang, Shaanxi, and Hunan provinces, respectively. Two cities and two counties were randomly selected in each province. Urban and suburban neighborhoods within the cities and townships and villages within the counties were selected randomly. In each community, all members meeting the inclusion and exclusion criteria of any of three nervous diseases in a randomly selected household were interviewed. The protocol of this study was reviewed and approved by the Institutional Review Board of the National Institute for Nutrition and Health (No. 2017020, 6 November 2017). Additionally, written informed consent was obtained for each participant before the survey.

The present study targeted subjects recruited in the cohort of AD. The samples eligible for inclusion were (1) 55 years old and older, (2) resident population living in the sampled community, (3) absence of clinically diagnosed AD, and (4) free of comorbid conditions that could affect assessment, such as congenital or acquired mental retardation, MCI, and visual/hearing abnormalities, even with correction. Subjects with completed data of sociodemographic characteristics, disease history, cognitive examination, food frequency questionnaire (FFQ), psychological evaluation, and survey of basic abilities of daily living were selected to participate in the present study. We excluded subjects because of their inability to perform basic activities of daily living involving eating, dressing, bathing, toileting, grooming, transferring to bed or chair, walking across a room, and urinary or fecal continence. Finally, a total of 4309 participants were involved in the analysis.

### 2.2. Assessment of Food Consumption

Dietary consumptions were assessed by a validated semi-quantitative FFQ covering 81 food items categorized as 13 major food groups and items in this study: rice, wheat, tubers, legumes, fresh vegetables, fresh fruit, pork, poultry, fish, beef or mutton, eggs, dairy, and nuts (Table S1). Participants were asked about the frequency of habitual consumption of each item during the last 12 months and chose among five categories of frequency (daily, weekly, monthly, annually, or never) and the amount consumed during the previous 12 months. For each item, if the participant was a non-consumer, then his/her consumption was set to zero grams daily or weekly. For consumers, their consumption of each food group or item was calculated by its reported average consumption frequency and quantity. Finally, the consumptions of rice, wheat, tubers, legumes, fresh vegetables, fresh fruit, pork, beef or mutton, dairy, and eggs were converted to daily grams, respectively, which were categorized into four levels by quartiles. The consumptions of fish, poultry, or nuts were grouped into four levels to reflect non-consumers for more than 25% of participants whose consumption of these three foods was zero, and tertiles of weekly consumption among corresponding consumers.

### 2.3. Assessment of Cognitive Function

The cognitive function of participants was evaluated using the Montreal Cognitive Assessment (MoCA). The MoCA included 52 items, of which scores of 32 items were calculated for the total MoCA scores ranging from 0 to 30 points which were positively associated with global cognitive function [24]. The criteria for MCI were according to Chinese MoCA norms [25]: total MoCA score ≤13 for illiterate individuals, ≤19 for individuals with 1 to 6 years of education, and ≤24 for those with 7 or more years of education.

The memory index score (MIS), executive index score (EIS), visuospatial index score (VIS), language index score (LIS), attention index score (AIS), and orientation index score (OIS) were applied to evaluate the cognitive domain function of memory, execution, visuospatial, language, attention, and orientation, respectively, and calculated based on the MoCA cognitive domain index score [24]. Participants who scored less than 1.5 SD below the age- and education-adjusted mean value in each cognitive domain were considered as being impaired in that cognitive domain [4]. Participants screened as having MCI and characterized by different cognitive domain deficits were categorized into 4 groups [26,27]: amnestic MCI single domain (aMCI-SD): only memory impairment; non-amnestic MCI single domain (naMCI-SD): a deficit in one cognitive domain other than memory; amnestic MCI multiple domains (aMCI-MD): memory impairment plus one other impaired domain; non-amnestic MCI multiple domains (naMCI-MD): deficits in at least 2 domains other than memory.

### 2.4. Assessment of Covariates

Interviewers with a degree in medicine or public health were required, and received two rounds of training conducted by national experts and provincial professionals, respectively. Then, those who passed a qualification test were appointed to use questionnaires to collect information on sociodemographic and health-related factors, including age, education (illiterate, ≤primary school, and ≥secondary school), resident area (rural, urban), current employment (no, yes), smoking (never, ever/current), alcohol intake (never, ever/current), monthly household income per capita (<1000, 1000–3999, ≥4000 (RMB)), physical activity, sleep duration, medical history, and medication use. Physical activity was assessed from four aspects: occupational, household chore, leisure time, and transportation activities. The intensity of total physical activity was assessed using metabolic equivalent of task (MET) hours per week based on the American College of Sports Medicine Association’s recommended standard [28], by tertiles (low, medium, and high), in the analysis. According to the National Sleep Foundation’s recommendations of sleep duration [29], the recommendation for the sleep duration of participants aged 55 to 64 years is 7–9 h, and for those aged ≥65 years it is 7–8 h. The individual total energy intake was summed up from all items in the FFQ linked to the China Food Composition Table [30].

Trained health workers measured individual waist circumference midway between the lowest rib and the iliac crest with a tape measure. Central obesity is defined as a waist circumference of ≥90 cm for men and ≥85 cm for women according to the criteria of weight for adults in China [31]. We used the Chinese body mass index (BMI) cutoff of 28 kg per square meter (kg/m^2^) to determine obesity [31]. Participants with a history of diet-related chronic diseases were defined as having hospital diagnosis of hypertension, diabetes, stroke, or myocardial infarction by professional doctors or receiving treatment for these diseases.

### 2.5. Statistical Analysis

Data were expressed as mean (SD) and *n* (%) for continuous variables and categorical variables, respectively. Differences in the prevalence of MCI and its subtypes of participants by different consumption levels of each food subgroup were analyzed by using a chi-square test. Differences in the distribution of global cognitive function score and cognitive domain subscores by different consumption levels of each food subgroup were examined using a Kruskal–Wallis H test. A series of multiple logistic regression models were conducted to assess the odds ratio (OR) and 95% confidence interval (CI) by levels of dietary consumption of each food item by adjusting for potential confounders, including demographics, socioeconomic status, lifestyle, energy intake, diet-related disease history, obesity, and central obesity. Quantile regression models were used to assess associations of food consumption with global cognitive score and cognitive domain subscores by adjusting for potential confounders. 

We conducted all statistical analyses using SAS version 9.4 (SAS Institute, Inc., Cary, NC, USA) and Stata version 12.0 (StataCorp., College Station, TX, USA). All statistical tests were two-tailed and considered significant at *p* < 0.05.

## 3. Results

### 3.1. Characteristics of Study Population

Among the 4309 participants included in the study, the mean age was 68.4 years (range 55 to 86), of which 80.1% were 55~74 years old; 54.6% were women, 49.4% were from urban areas, 76.4% had a monthly household income per capita of more than RMB 1000, 85.8% of the subjects had a primary or above education level, and less than 40% of participants had histories of diet-related chronic diseases (Table S2). Overall, participants tended to consume rice, wheat, fresh vegetables, and pork, and close to 25% of them reported to not consume beef or mutton, poultry, fish, eggs, dairy, or nuts (Table 1).

### 3.2. Cognitive Function by Demographics and Health-Related Factors

Six hundred and five participants were found to have MCI, resulting in a prevalence of 42.6%. The prevalence of each subtype of MCI was less than 10%, of which the highest one was 8.2%, observed in the group with aMCI-MD. Compared to normal cognition participants, those with MCI or its subtypes tended to be older, have a lower monthly income, live in a rural area, and have other health-related problems (Table S2). 

The average score of global cognitive function among participants was 21.53, and each cognitive domain score was more than half of the total score of the corresponding subdomain. The scores of global cognitive function and cognitive domains were strongly significantly different by age group, education level, residential area, monthly income, and health-related factors. Generally, the older population, women, and those with a lower education level and a worse economic or health state had lower scores of global cognitive function and cognitive domains, compared to those of their counterparts (Table S3).

### 3.3. Association of Food Consumptions with MCI and Its Subtypes

The prevalence of MCI and its subtypes in the bottom quartile consumption level of rice was significantly higher than that of other rice consumption levels, while among all wheat consumption levels, the highest prevalence of MCI, naMCI-SD, aMCI-MD, and naMCI-MD was observed in its top quartile consumption level. The prevalence of MCI in the highest consumption level of legumes, fresh vegetables, pork, poultry, beef of mutton, fish, or nuts was obviously lower as compared with that of other consumption levels of the corresponding food. Moreover, participants with one of the MCI subtypes tended to cluster in the lower consumption levels of fresh vegetables and fish, and those with aMCI-MD and naMCI-MD consumed less of legumes, pork, and nuts (Table 2).

Table 3 shows the odds ratio (95% CI) between the consumption level of food items and MCI and its subtypes in Chinese people aged above 55 years old. After adjusting for potential factors, participants in the top quartile level of rice, legumes, fresh vegetables, fresh fruit, pork, beef or mutton, poultry, fish, and nuts had lower ORs of MCI, whereas those with the highest consumption level of wheat and egg had higher ORs of MCI in comparison to their bottom level (*p* < 0.05). In addition, compared to the first quartile of consumption, participants in the second quartile level of beef or mutton, poultry, and fish had 89% (OR = 1.89, 95%CI: 1.18~3.02), 64% (OR = 1.64, 95%CI: 1.10~2.45), and 82% (OR = 1.82, 95%CI: 1.19~2.78) higher odds of aMCI-SD, respectively, while those in higher consumption levels of these foods were inversely associated with ORs of other subtypes of MCI. As compared with non-consumers, dairy or nut consumers had higher odds of aMCI-SD and aMCI-MD and, inversely, those with higher consumption of these foods were associated with decreased odds of naMCI-SD and naMCI-MD.

### 3.4. Association between Food Consumptions and Cognitive Domains

The lowest scores of global cognitive function and cognitive domains were observed in the bottom consumption level of rice, legumes, fresh vegetables, fresh fruit, pork, and fish, and in the top consumption level of wheat, among the four consumption levels of the above foods (*p* < 0.001). Moreover, participants who consumed dairy had higher scores of global cognitive function and indexes of cognitive domains other than memory compared to the non-consumers (*p* < 0.001) (Table 4).

As compared with the bottom consumption level, participants with higher consumption of rice had higher scores of global cognitive function and indexes of cognitive domains, while those with higher consumption of wheat, ranging from the second quartile level to the top level, had lower scores of global cognitive function and EIS (*p* < 0.05) after adjusting for potential factors. The score of global cognitive function, EIS, VIS, and LIS were positively associated with the higher consumption levels of legumes, ranging from the second quartile consumption level to the top level, in comparison to the bottom one (*p* < 0.05). In addition, the scores of global cognitive function and indexes of several types of cognitive domains increased in the top consumption level of fresh vegetables, fresh fruit, pork, poultry, fish, and nuts, compared to the bottom counterpart. No significant association was observed between OIS and an increased consumption level of each selected food in comparison to the corresponding bottom counterpart (*p* > 0.05) (Table 5).

## 4. Discussion

In our study involving four provincial men and women aged 55 years and above in China without prior AD or related medication for mental disease, we observed that the participants’ cognition function, including MCI and its subtypes, as well as global cognitive function and specific domains, varied with different consumption levels of the main selected foods. Moreover, increased consumption levels of rice, legumes, fresh vegetables, and pork were associated with better cognitive function, whereas the inverse association was observed between wheat and egg consumption and cognition. 

The prevalence of MCI, ranging from 3.2% to 32.6%, varied in populations of elderly Americans, Finns, French, and Swedish [32,33,34,35], and was lower than that of individuals included in this study. This, to some extent, reflects the severe situation of cognition decline among the Chinese population even with differences in the range of participants’ ages or the diagnosis criteria of MCI. The MCI progression factors were characterized as older age and worse income level [36]. Similarly, a finding from our study showed that participants with MCI and its subtypes clustered with old age, rural area, and lower monthly income level groups. Previous studies reported that the prevalence of amnestic MCI was significantly higher than that of non-amnestic MCI [37,38], suggesting that MCI with memory impairment was a more common MCI subtype. However, Jungwirth and coworkers reported that the percentage of non-amnestic MCI was 6% higher than that of amnestic MCI (14.86% vs. 9.0%) among 592 Australians at age 75 to 76 years old [39], and Busse et al. [40] showed no obvious difference in the prevalence of these two subtypes, which was similar to our results, just with a prevalence difference of 0.1%. Accordingly, there are inconsistent results in identifying the dominant subtype of MCI. 

Epidemiological evidence supported the hypothesis that the diet disparity was significant between normal cognition individuals and MCI ones [21,23], and indicated that a diet characterized by high consumption of fruit, vegetables, nuts, legumes, fish, and low consumption of red meat and poultry, was associated with a decreased risk of cognitive decline [41]. Further, special attention has been devoted to the consumption of fruit, vegetables, fish, and nuts because of their nutrient profiles that are rich in antioxidants or polyunsaturated fatty acids, which are considered to have anti-inflammatory properties, while oxidative stress and inflammation are incentive factors of the cognitive decline process [42]. A cross-sectional study conducted with 1849 Brazilian subjects with an average age of 77.5 years old showed that a daily consumption of vegetables and fruit ≥400 g was associated with a decreased prevalence of cognitive impairment (OR = 0.53, 95% CI: 0.31~0.89). O’Brien et al. followed up 15,467 women aged 70 or older for 6 years and observed that those with a higher long-term nut intake (>5 servings of nuts/week) had a significantly higher score of global cognitive function than that of non-consumers [43]. In the Chinese population, adults with a daily nut consumption of more than 10 g had 40% decreased odds of poor cognitive function (OR = 0.60, 95%CI: 0.43~0.84) among the sample of 4822 participants aged 55 and over [44]; those aged 65 years old and over with at least 1 serving/week of fish tended to have a better global cognitive function, found in a prospective cohort study with a follow-up of 5.3 years of 1566 participants [22]. Generally, the frequent consumption of fruit, vegetables, fish, and nuts was related to relatively healthy cognitive function, which was similar to results from our study that increased consumption of these foods decreased the odds of MCI and some of its subtypes, as well as was positively associated with higher scores of extensive cognitive domains. However, no significant association was found between the consumption of fish, fruit, and vegetables and the risk of cognitive impairment among elderly French people from the Three-City Study [8], as well as others [45,46]. The disparities in populations with distinctive diets might be a potential explanation for these discrepancies. Indeed, it was neglected that there are regional disparities in the subgroups of each food item. 

For other food items, meat consumption was generally considered to have an adverse effect on cognitive outcomes due to its high saturated fat content [47,48] which is potentially associated with the overproduction of circulating free fatty acids and systemic inflammation. However, the specific effect of meat consumption on cognitive disorders was often discordant [18]. A longitudinal study of a large cohort with a mean follow-up of 9.8 years and active ascertainment of dementia showed that low meat consumption (≤1 time/week) was associated with an increased risk of cognitive impairment compared with regular consumption (≥4 times/week) [8]. Another study observed a positive association of red meat with entorhinal cortex thickness, which was negatively related to dementia [49]. In our study, we observed that eating adequately varied meat, including pork, beef or mutton, and poultry, was positively associated with a better comprehensive cognitive state. Given that lean meat and poultry are high in protein, which is related to superior cognitive function [50], moderation of meat consumption is probably advised due to the controversial association between meat consumption and cognition, along with the potential risks of overweight and obesity. 

A stable blood glucose profile is associated with better cognitive function and a lower risk of cognitive impairments [51]. In general, food with a low glycemic index and low glycemic load (e.g., vegetables, legumes, and whole grains) is less likely to detrimentally impact glucose metabolism and neuronal integrity. Rice and wheat are both acknowledged for their high carbohydrate content and glycemic index, and we found that participants who preferred to consume wheat, mainly as low-fiber wheat products including non-fried noodles, white bread, steamed buns, dumplings, etc., had relatively worse cognitive function and we unexpectedly observed that higher rice consumption was associated with decreased odds of MCI and aMCI-SD in comparison with the bottom level, as well as with better function of most cognitive domains in the current study. Of note, the beneficial effect was seen at a moderate consumption level of the third quartile rather than the top one. Kim et al. [52] also found, among Korean adults aged over 50 years in 2018, that a moderate consumption of cooked white rice was negatively associated with the risk of MCI, adding data supporting a positive link between cognition and rice consumption within a considerable range. Previous studies showed the different associations of rice intake and wheat intake with metabolic syndrome, diabetes, and dyslipidemia among the Chinese population [53,54]. However, few studies have been conducted in the field of the rice–cognition and wheat–cognition relationship. Given the cross-sectional nature of our study, large-scale prospective cohort studies are required to provide stronger evidence. Overall, moderation of rice consumption may be necessary, although the threshold–effect association remains unclear, and further research on it, of course, is required to elicit the potential mechanism in order to identify the optimal recommendations. 

This study is the first to separately examine the associations between food consumptions and each subtype of MCI and multiple cognitive domains. We found the selected foods have similar links to various cognitive domains, consistently positive or negative, and a similar relationship of most these foods among subtypes of MCI, but not completely. For instance, the highest consumption level of dairy decreased the prevalence of naMCI-SD or naMCI-MD, whereas it was associated with 50% higher odds of aMCI-SD. This observation suggests that it is favorable to differentiate different subtypes of MCI when identifying the impact of exposures, like dietary factors, on MCI due to differentially regional impairment features of diverse subtypes [55,56,57]. Only presenting the relationship of exposures, like dietary factors, with MCI might lead to controversy for the potential interactions among distinct subtypes. Apart from this, various MCI subtypes have been proposed to broaden the concept of the pathology of different subtypes of MCI. Given that the transition probabilities from the MCI subtypes with memory impairment to AD were reported to be higher [58], to focus on isolated subtypes of MCI may add value for developing accurate strategies to combat AD. 

Our study has several limitations. First, the dietary consumption level was estimated based on an FFQ that covered the past 12 months, which may lead to a recall bias. Second, the relative precision of the estimation of dietary consumption level relied on the self-reported exposure information from people with normal cognition, thus, the reliance on cognitive ability of the FFQ method may not have led to a precise estimate of dietary intake in all similar prospective epidemiological studies. Recall error due to cognitive impairment is thought to bias results towards the null hypothesis [59], nevertheless, we did sensitivity analyses in which we excluded those with the lowest 5% scores of the MoCA and found associations consistent with those modeled by multiple logistic regression. Third, a huge proportion of participants did not consume nuts and dairy, and the consumption of them was relatively low, so we could not clarify the appropriate dose in the association analysis and had difficulty in ranking their consumption. Fourth, although we adjusted carefully for some covariates during the data analysis, residual confounding was still possible. In addition, the cross-sectional nature of our study does not allow us to draw any causal conclusions. The major strengths of this study include the use of the MoCA to evaluate overall cognitive function and domains and recognize the subtypes of MCI, and the use of a relatively unlimited population-based sample from CCSNSD, which imparts the ability to generalize the results to the Chinese population in part.

## 5. Conclusions

In conclusion, most diet global cognitive benefits were observed to be associated with the preexisting consumption of foods in the present study, and adaptation to a higher consumption of rice, legumes, fresh vegetables, fresh fruit, meat, and nuts may be primarily considered as the benefits. Additionally, this study has revealed the heterogeneity of associations between the consumption of certain foods and MCI subtypes, representing value in developing accurate strategies against the progress of cognitive impairment. Further studies, including more cohort studies or randomized clinical trials, are needed to confirm these observations.

## Figures and Tables

**Table 1:** Food consumption levels of Chinese adults aged 55 years and above in four provinces in CCSNSD 2018–2019 ^a^.

Foods	Q1/T0	Q2/T1	Q3/T2	Q4/T3
Rice (g/day)	10.0 (3.3, 21.4)	74.6 (45.7, 100.0)	180.0 (150.0, 200.0)	300.0 (300.0, 450.0)
Wheat (g/day)	7.1 (0.0, 14.3)	50.0 (40.0, 60.0)	100.0 (100.0, 120.0)	300.0 (200.0, 360.0)
Tubers (g/day)	0.0 (0.0, 3.9)	13.1 (9.3, 15.0)	28.6 (24.8, 35.0)	67.1 (51.4, 100.0)
Legumes (g/day)	3.3 (0.0, 7.1)	20.0 (14.3, 25.4)	44.2 (37.1, 57.1)	113.1 (85.7, 158.9)
Fresh vegetables (g/day)	47.4 (30.0, 63.6)	117.0 (98.4, 140.0)	228.0 (195.6, 259.9)	408.2 (342.5, 530.2)
Fresh fruit (g/day)	6.7 (0.0, 11.4)	27.1 (21.5, 33.2)	57.5 (48.7, 70.8)	144.3 (107.1, 205.7)
Pork (g/day)	2.7 (0.0, 5.0)	14.3 (11.5, 17.9)	32.9 (28.6, 42.9)	100.0 (71.4, 161.0)
Beef or mutton (g/day)	0.0 (0.0, 0.0)	0.5 (0.3, 0.7)	2.5 (1.6, 3.3)	10.0 (8.0, 16.7)
Poultry (g/week)	0.0 (0.0, 0.0)	18.4 (11.7, 23.3)	50.0 (46.7, 70.0)	150.0 (100.0, 220.0)
Fish (g/week)	0.0 (0.0, 0.0)	23.3 (11.7, 28.0)	80.0 (51.5, 100.0)	300.0 (200.0, 432.0)
Eggs (g/day)	6.3 (0.0, 8.7)	20.0 (15.4, 21.7)	42.9 (32.0, 50.0)	60.5 (60.0, 81.8)
Dairy (g/day)	0.0 (0.0, 0.0)	21.4 (8.6, 31.2)	80.0 (57.5, 100.0)	206.3 (163.8, 257.1)
Nuts (g/week)	0.0 (0.0, 0.0)	5.8 (2.9, 9.6)	23.3 (18.7, 42.0)	140.0 (93.3, 210.0)

^a^: expressed as median (P25, p75). Q1–Q4 are consumption levels of foods (except dairy and nuts) grouped by quartile consumption; T0 = non-consumer group for dairy and nuts, T1–T3 are consumption levels of dairy and nuts grouped by the tertile consumption of consumers.

**Table 2:** Prevalence of MCI and its subtypes of participants according to consumption levels of food subgroups **^a^**.

Foods	MCI		*p* Value	MCI Subtypes				*p* Value
	Yes	No		aMCI-SD	naMCI-SD	aMCI-MD	naMCI-MD	
Rice			<0.001					0.005
Q1	586 (51.0)	564 (49.0)		73 (6.3)	90 (7.8)	127 (11.0)	91 (7.9)	
Q2	504 (44.8)	620 (55.2)		53 (4.7)	76 (6.8)	75 (6.7)	54 (4.8)	
Q3	374 (36.3)	657 (63.7)		37 (3.6)	65 (6.3)	61 (5.9)	75 (7.3)	
Q4	370 (36.9)	634 (63.1)		42 (4.2)	66 (6.6)	92 (9.2)	39 (3.9)	
Wheat			<0.001					0.045
Q1	330 (31.6)	713 (68.4)		32 (3.1)	55 (5.3)	78 (7.5)	59 (5.7)	
Q2	479 (44.4)	600 (55.6)		47 (4.4)	72 (6.7)	75 (7.0)	49 (4.5)	
Q3	495 (44.9)	607 (55.1)		72 (6.5)	68 (6.2)	85 (7.7)	64 (5.8)	
Q4	530 (48.8)	555 (51.2)		54 (5.0)	102 (9.4)	117 (10.8)	87 (8.0)	
Tubers			0.698					<0.001
Q1	444 (42.0)	613 (58.0)		33 (3.1)	74 (7.0)	95 (9.0)	101 (9.6)	
Q2	455 (41.6)	638 (58.4)		60 (5.5)	76 (7.0)	93 (8.5)	54 (4.9)	
Q3	473 (44.0)	602 (56.0)		54 (5.0)	72 (6.7)	87 (8.1)	52 (4.8)	
Q4	462 (42.6)	622 (57.4)		58 (5.4)	75 (6.9)	80 (7.4)	52 (4.8)	
Legumes			<0.001					<0.001
Q1	533 (50.1)	531 (49.9)		47 (4.4)	86 (8.1)	149 (14.0)	105 (9.9)	
Q2	468 (42.8)	625 (57.2)		63 (5.8)	78 (7.1)	104 (9.5)	63 (5.8)	
Q3	438 (40.7)	637 (59.3)		41 (3.8)	74 (6.9)	59 (5.5)	55 (5.1)	
Q4	395 (36.7)	682 (63.3)		54 (5.0)	59 (5.5)	43 (4.0)	36 (3.3)	
Fresh vegetables			<0.001					0.007
Q1	504 (46.8)	573 (53.2)		68 (6.3)	82 (7.6)	121 (11.2)	75 (7.0)	
Q2	513 (47.6)	564 (52.4)		53 (4.9)	73 (6.8)	119 (11.0)	74 (6.9)	
Q3	448 (41.6)	629 (58.4)		46 (4.3)	72 (6.7)	69 (6.4)	69 (6.4)	
Q4	369 (34.2)	709 (65.8)		38 (3.5)	70 (6.5)	46 (4.3)	41 (3.8)	
Fresh fruit			<0.001					0.004
Q1	536 (49.8)	540 (50.2)		45 (4.2)	99 (9.2)	133 (12.4)	96 (8.9)	
Q2	492 (45.8)	582 (54.2)		62 (5.8)	69 (6.4)	95 (8.8)	62 (5.8)	
Q3	384 (35.4)	700 (64.6)		41 (3.8)	59 (5.4)	54 (5.0)	57 (5.3)	
Q4	422 (39.3)	653 (60.7)		57 (5.3)	70 (6.5)	73 (6.8)	44 (4.1)	
Pork			<0.001					0.009
Q1	546 (50.3)	540 (49.7)		76 (7.0)	83 (7.6)	118 (10.9)	69 (6.4)	
Q2	482 (45.1)	586 (54.9)		51 (4.8)	86 (8.1)	75 (7.0)	78 (7.3)	
Q3	404 (37.6)	671 (62.4)		35 (3.3)	59 (5.5)	99 (9.2)	58 (5.4)	
Q4	402 (37.2)	678 (62.8)		43 (4.0)	69 (6.4)	63 (5.8)	54 (5.0)	
Beef or mutton			<0.001					<0.001
Q1	758 (46.5)	872 (53.5)		59 (3.6)	123 (7.5)	173 (10.6)	141 (8.7)	
Q2	243 (46.6)	278 (53.4)		37 (7.1)	29 (5.6)	68 (13.1)	26 (5.0)	
Q3	442 (40.9)	640 (59.1)		72 (6.7)	66 (6.1)	71 (6.6)	44 (4.1)	
Q4	391 (36.3)	685 (63.7)		37 (3.4)	79 (7.3)	43 (4.0)	48 (4.5)	
Poultry			<0.001					0.063
Q1	534 (49.4)	548 (50.6)		53 (4.9)	83 (7.7)	114 (10.5)	85 (7.9)	
Q2	503 (46.9)	570 (53.1)		74 (6.9)	80 (7.5)	114 (10.6)	67 (6.2)	
Q3	420 (40.4)	620 (59.6)		42 (4.0)	79 (7.6)	65 (6.3)	50 (4.8)	
Q4	377 (33.8)	737 (66.2)		36 (3.2)	55 (4.9)	62 (5.6)	57 (5.1)	
Fish			<0.001					<0.001
Q1	522 (48.5)	555 (51.5)		42 (3.9)	100 (9.3)	107 (9.9)	79 (7.3)	
Q2	527 (49.6)	535 (50.4)		78 (7.3)	62 (5.8)	126 (11.9)	52 (4.9)	
Q3	416 (38.7)	658 (61.3)		45 (4.2)	74 (6.9)	67 (6.2)	50 (4.7)	
Q4	369 (33.7)	727 (66.3)		40 (3.6)	61 (5.6)	55 (5.0)	78 (7.1)	
Eggs			0.007					0.018
Q1	415 (38.5)	664 (61.5)		44 (4.1)	56 (5.2)	102 (9.5)	64 (5.9)	
Q2	480 (43.3)	629 (56.7)		48 (4.3)	99 (8.9)	79 (7.1)	58 (5.2)	
Q3	447 (42.7)	599 (57.3)		50 (4.8)	69 (6.6)	76 (7.3)	66 (6.3)	
Q4	492 (45.8)	583 (54.2)		63 (5.9)	73 (6.8)	98 (9.1)	71 (6.6)	
Dairy			0.242					<0.001
T0	1049 (43.2)	1378 (56.8)		99 (4.1)	172 (7.1)	223 (9.2)	187 (7.7)	
T1	289 (44.2)	365 (55.8)		38 (5.8)	54 (8.3)	63 (9.6)	27 (4.1)	
T2	236 (39.2)	366 (60.8)		21 (3.5)	43 (7.1)	31 (5.1)	26 (4.3)	
T3	260 (41.5)	366 (58.5)		47 (7.5)	28 (4.5)	38 (6.1)	19 (3.0)	
Nuts			<0.001					<0.001
T0	874 (46.8)	993 (53.2)		54 (2.9)	151 (8.1)	166 (8.9)	140 (7.5)	
T1	376 (46.2)	438 (53.8)		72 (8.8)	34 (4.2)	96 (11.8)	48 (5.9)	
T2	317 (38.8)	501 (61.2)		44 (5.4)	59 (7.2)	51 (6.2)	45 (5.5)	
T3	267 (33.0)	543 (67.0)		35 (4.3)	53 (6.5)	42 (5.2)	26 (3.2)	

^a^: expressed as the number of subjects for each category (%). Q1–Q4 are consumption levels of foods (except dairy and nuts) grouped by their quartile consumption; T0 = non-consumer group for dairy and nuts, T1–T3 are consumption levels of dairy and nuts grouped by the tertile consumption of consumers. *p* value < 0.05 was considered to be statistically significant, examined by chi-square test.

**Table 3:** Associations of food consumption with MCI and its subtypes using multiple logistic regression model ^a^.

Foods	MCI	MCI Subtypes			
		aMCI-SD	naMCI-SD	aMCI-MD	naMCI-MD
Rice					
Q2	0.87 (0.73, 1.05)	0.69 (0.45, 1.06)	0.90 (0.62, 1.29)	0.93 (0.66, 1.31)	0.86 (0.58, 1.27)
Q3	0.65 (0.54, 0.78) ^†^	0.47 (0.30, 0.75) ^†^	0.69 (0.48, 1.00)	0.70 (0.49, 1.01)	1.05 (0.73, 1.50)
Q4	0.83 (0.68, 1.00) ^†^	0.69 (0.45, 1.08)	0.94 (0.65, 1.37)	1.25 (0.90, 1.76)	0.74 (0.48, 1.15)
Wheat					
Q2	1.52 (1.26, 1.83) ^†^	1.35 (0.83, 2.19)	1.46 (0.99, 2.15)	1.09 (0.76, 1.57)	0.88 (0.58, 1.34)
Q3	1.43 (1.18, 1.73) ^†^	1.84 (1.16, 2.91) ^†^	1.15 (0.78, 1.72)	1.25 (0.87, 1.80)	1.08 (0.72, 1.62)
Q4	1.52 (1.25, 1.85) ^†^	1.41 (0.86, 2.32)	1.89 (1.29, 2.77) ^†^	1.06 (0.75, 1.51)	1.19 (0.79, 1.77)
Tubers					
Q2	0.93 (0.77, 1.12)	1.61 (1.02, 2.55) ^†^	0.99 (0.69, 1.41)	0.99 (0.71, 1.38)	0.56 (0.39, 0.81) ^†^
Q3	1.02 (0.85, 1.22)	1.42 (0.89, 2.26)	0.96 (0.67, 1.38)	0.98 (0.70, 1.38)	0.53 (0.36, 0.77) ^†^
Q4	1.03 (0.85, 1.23)	1.57 (0.99, 2.52)	1.12 (0.78, 1.62)	0.79 (0.56, 1.12)	0.52 (0.36, 0.77) ^†^
Legumes					
Q2	0.72 (0.60, 0.86) ^†^	0.91 (0.60, 1.38)	0.71 (0.50, 1.01)	0.71 (0.53, 0.96) ^†^	0.59 (0.41, 0.84) ^†^
Q3	0.68 (0.57, 0.82) ^†^	0.63 (0.39, 0.99) ^†^	0.71 (0.50, 1.02)	0.49 (0.34, 0.69) ^†^	0.61 (0.42, 0.88) ^†^
Q4	0.57 (0.48, 0.69) ^†^	0.75 (0.48, 1.17)	0.55 (0.38, 0.80) ^†^	0.35 (0.24, 0.51) ^†^	0.37 (0.25, 0.57) ^†^
Fresh vegetables					
Q2	1.03 (0.86, 1.23)	0.72 (0.48, 1.08)	0.94 (0.66, 1.34)	1.06 (0.78, 1.43)	1.13 (0.79, 1.62)
Q3	0.88 (0.73, 1.06)	0.60 (0.39, 0.91) ^†^	0.96 (0.67, 1.37)	0.69 (0.49, 0.97) ^†^	1.10 (0.76, 1.59)
Q4	0.66 (0.54, 0.80)^†^	0.39 (0.25, 0.62) ^†^	0.83 (0.58, 1.20)	0.46 (0.31, 0.67) ^†^	0.69 (0.45, 1.06)
Fresh fruit					
Q2	0.93 (0.77, 1.11)	1.58 (1.02, 2.45) ^†^	0.72 (0.51, 1.02)	0.89 (0.65, 1.23)	0.73 (0.51, 1.05)
Q3	0.56 (0.47, 0.68) ^†^	0.77 (0.48, 1.25)	0.51 (0.35, 0.73) ^†^	0.49 (0.34, 0.70) ^†^	0.64 (0.44, 0.92) ^†^
Q4	0.71 (0.58, 0.85) ^†^	1.14 (0.72, 1.81)	0.64 (0.45, 0.92) ^†^	0.88 (0.62, 1.24)	0.62 (0.41, 0.93) ^†^
Pork					
Q2	0.83 (0.70, 1.00) ^†^	0.61 (0.41, 0.91) ^†^	1.03 (0.73, 1.45)	0.68 (0.49, 0.96) ^†^	1.16 (0.80, 1.67)
Q3	0.68 (0.56, 0.82) ^†^	0.39 (0.25, 0.61) ^†^	0.73 (0.50, 1.06)	0.93 (0.67, 1.29)	0.92 (0.62, 1.36)
Q4	0.74 (0.61, 0.89) ^†^	0.48 (0.32, 0.74) ^†^	0.93 (0.64, 1.35)	0.68 (0.48, 0.98) ^†^	1.02 (0.68, 1.54)
Beef or mutton					
Q2	0.99 (0.81, 1.23)	1.89 (1.18, 3.02) ^†^	0.79 (0.50, 1.24)	1.18 (0.84, 1.65)	0.56 (0.35, 0.89) ^†^
Q3	0.80 (0.67, 0.94) ^†^	1.57 (1.07, 2.32) ^†^	0.75 (0.53, 1.05)	0.74 (0.54, 1.02)	0.50 (0.34, 0.73) ^†^
Q4	0.74 (0.62, 0.88) ^†^	0.79 (0.50, 1.25)	0.99 (0.71, 1.37)	0.54 (0.37, 0.79) ^†^	0.69 (0.47, 1.00)
Poultry					
Q2	0.94 (0.79, 1.12)	1.64 (1.10, 2.45) ^†^	0.98 (0.69, 1.39)	1.01 (0.74, 1.38)	0.74 (0.51, 1.07)
Q3	0.81 (0.67, 0.97) ^†^	0.82 (0.52, 1.29)	1.05 (0.73, 1.49)	0.85 (0.59, 1.21)	0.75 (0.51, 1.12)
Q4	0.62 (0.52, 0.75) ^†^	0.59 (0.37, 0.94) ^†^	0.61 (0.41, 0.89) ^†^	0.67 (0.47, 0.96) ^†^	0.72 (0.49, 1.06)
Fish					
Q2	1.01 (0.84, 1.21)	1.82 (1.19, 2.78) ^†^	0.68 (0.47, 0.97) ^†^	1.35 (0.99, 1.84)	0.70 (0.47, 1.04)
Q3	0.72 (0.60, 0.87) ^†^	0.91 (0.57, 1.46)	0.74 (0.52, 1.05)	0.79 (0.56, 1.13)	0.72 (0.48, 1.07)
Q4	0.68 (0.56, 0.82) ^†^	0.76 (0.47, 1.24)	0.65 (0.45, 0.94) ^†^	0.60 (0.41, 0.87) ^†^	1.10 (0.76, 1.59)
Eggs					
Q2	1.15 (0.96, 1.38)	1.10 (0.70, 1.70)	1.78 (1.25, 2.56) ^†^	0.93 (0.67, 1.31)	1.00 (0.67, 1.47)
Q3	1.05 (0.88, 1.26)	1.01 (0.65, 1.57)	1.26 (0.86, 1.85)	0.81 (0.57, 1.14)	1.14 (0.78, 1.68)
Q4	1.23 (1.03, 1.48) ^†^	1.48 (0.97, 2.27)	1.57 (1.07, 2.31) ^†^	1.08 (0.78, 1.50)	1.32 (0.90, 1.94)
Dairy					
T1	1.08 (0.89, 1.30)	1.54 (1.01, 2.35) ^†^	1.21 (0.86, 1.72)	1.47 (1.05, 2.05) ^†^	0.69 (0.45, 1.08)
T2	0.82 (0.67, 1.00) ^†^	0.75 (0.45, 1.26)	0.86 (0.59, 1.25)	0.93 (0.60, 1.42)	0.67 (0.42, 1.06)
T3	0.87 (0.72, 1.07)	1.51 (1.00, 2.29) ^†^	0.60 (0.38, 0.93) ^†^	1.09 (0.73, 1.63)	0.49 (0.29, 0.82) ^†^
Nuts					
T1	1.00 (0.84, 1.20)	3.01 (2.03, 4.47) ^†^	0.51 (0.34, 0.77) ^†^	1.63 (1.20, 2.21) ^†^	0.87 (0.60, 1.26)
T2	0.77 (0.64, 0.92)	1.69 (1.09, 2.62) ^†^	0.83 (0.59, 1.17)	0.87 (0.60, 1.24)	0.79 (0.54, 1.16)
T3	0.65 (0.54, 0.78) ^†^	1.40 (0.87, 2.24)	0.77 (0.54, 1.09)	0.80 (0.55, 1.17)	0.48 (0.30, 0.75) ^†^

^a^: expressed as OR (95% CI). Q = quartile, reference = Q1; T = tertile, reference = T0 (non-consumer). Adjusted for age, gender, residential area, education level, current employment, income level, physical activity, smoking, alcohol intake, sleep duration status, energy, disease history, obesity, and central obesity. ^†^: *p* value < 0.05.

**Table 4:** Global cognitive function score and cognitive domain subscores of participants according to consumption levels of food subgroups ^a^.

Foods	Global Cognitive Function	Cognition Domain Scores					
		MIS	EIS	VIS	LIS	AIS	OIS
Rice							
Q1	19.55 ± 6.17	10.00 ± 4.59	7.61 ± 3.34	4.97 ± 1.75	4.36 ± 1.35	12.09 ± 4.31	5.23 ± 1.22
Q2	22.03 ± 5.74	11.19 ± 4.00	9.04 ± 3.13	5.49 ± 1.56	4.59 ± 1.36	13.19 ± 3.95	5.57 ± 0.92
Q3	22.68 ± 6.44	11.80 ± 3.95	9.17 ± 3.45	5.44 ± 1.85	4.65 ± 1.45	13.90 ± 3.85	5.60 ± 0.88
Q4	22.05 ± 6.22	11.40 ± 4.09	9.06 ± 3.24	5.17 ± 1.91	4.40 ± 1.50	13.59 ± 3.69	5.59 ± 0.84
Wheat							
Q1	22.71 ± 6.63	11.77 ± 4.02	9.28 ± 3.51	5.40 ± 1.90	4.54 ± 1.51	13.67 ± 4.00	5.60 ± 0.87
Q2	21.95 ± 5.76	11.18 ± 3.89	8.92 ± 3.13	5.41 ± 1.70	4.59 ± 1.37	13.38 ± 3.77	5.63 ± 0.81
Q3	21.82 ± 5.92	11.09 ± 4.23	8.88 ± 3.20	5.31 ± 1.74	4.56 ± 1.39	13.39 ± 3.90	5.59 ± 0.92
Q4	19.68 ± 6.31	10.27 ± 4.60	7.71 ± 3.38	4.95 ± 1.74	4.30 ± 1.40	12.22 ± 4.27	5.15 ± 1.24
Tubers							
Q1	21.07 ± 6.74 *	11.07 ± 4.22 *	8.45 ± 3.61 *	5.08 ± 1.93	4.33 ± 1.50	12.74 ± 4.22	5.38 ± 1.08
Q2	21.81 ± 6.28	11.24 ± 4.19	8.85 ± 3.33	5.35 ± 1.76	4.56 ± 1.44	13.35 ± 3.89	5.51 ± 0.97
Q3	21.74 ± 6.10	11.03 ± 4.30	8.79 ± 3.27	5.35 ± 1.72	4.60 ± 1.35	13.28 ± 4.01	5.55 ± 0.95
Q4	21.49 ± 5.88	10.93 ± 4.20	8.68 ± 3.19	5.28 ± 1.69	4.51 ± 1.38	13.25 ± 3.97	5.53 ± 0.97
Legumes							
Q1	19.47 ± 6.68	9.94 ± 4.67	7.74 ± 3.52	4.70 ± 1.95	4.08 ± 1.52	11.95 ± 4.34	5.26 ± 1.19
Q2	21.44 ± 6.36	10.98 ± 4.41	8.64 ± 3.42	5.25 ± 1.73	4.57 ± 1.40	13.16 ± 4.06	5.47 ± 1.02
Q3	22.16 ± 5.88	11.52 ± 3.78	9.01 ± 3.20	5.50 ± 1.64	4.57 ± 1.38	13.44 ± 3.81	5.54 ± 0.91
Q4	23.02 ± 5.50	11.82 ± 3.74	9.39 ± 3.05	5.61 ± 1.64	4.77 ± 1.29	14.08 ± 3.57	5.70 ± 0.76
Fresh vegetables							
Q1	20.37 ± 6.29	10.28 ± 4.62	8.16 ± 3.28	5.05 ± 1.75	4.30 ± 1.48	12.35 ± 4.11	5.39 ± 1.12
Q2	20.61 ± 6.45	10.52 ± 4.39	8.24 ± 3.49	5.14 ± 1.82	4.38 ± 1.43	12.53 ± 4.26	5.41 ± 1.08
Q3	21.91 ± 6.18	11.44 ± 3.94	8.88 ± 3.35	5.31 ± 1.76	4.55 ± 1.40	13.49 ± 3.82	5.48 ± 0.97
Q4	23.23 ± 5.67	12.04 ± 3.67	9.49 ± 3.13	5.55 ± 1.74	4.77 ± 1.32	14.27 ± 3.60	5.69 ± 0.75
Fresh fruit							
Q1	19.62 ± 6.48	10.31 ± 4.56	7.70 ± 3.43	4.78 ± 1.89	4.15 ± 1.54	12.08 ± 4.22	5.25 ± 1.16
Q2	21.02 ± 6.01	10.73 ± 4.18	8.46 ± 3.25	5.11 ± 1.77	4.43 ± 1.39	12.96 ± 3.87	5.51 ± 0.97
Q3	22.66 ± 6.05	11.66 ± 3.86	9.28 ± 3.26	5.54 ± 1.71	4.68 ± 1.37	13.71 ± 3.87	5.59 ± 0.93
Q4	22.81 ± 5.94	11.58 ± 4.14	9.34 ± 3.21	5.63 ± 1.60	4.74 ± 1.29	13.87 ± 3.89	5.63 ± 0.86
Pork							
Q1	19.98 ± 6.11	10.17 ± 4.57	7.97 ± 3.23	5.04 ± 1.76	4.28 ± 1.46	12.24 ± 4.20	5.32 ± 1.15
Q2	21.30 ± 6.29	11.02 ± 4.05	8.60 ± 3.38	5.22 ± 1.79	4.49 ± 1.45	12.96 ± 3.98	5.44 ± 1.07
Q3	22.18 ± 6.35	11.19 ± 4.26	9.02 ± 3.43	5.42 ± 1.76	4.59 ± 1.38	13.52 ± 4.04	5.58 ± 0.91
Q4	22.66 ± 5.96	11.89 ± 3.81	9.18 ± 3.25	5.38 ± 1.78	4.63 ± 1.37	13.92 ± 3.68	5.64 ± 0.78
Beef or mutton							
Q1	20.38 ± 6.61	10.65 ± 4.52	8.03 ± 3.50	5.03 ± 1.83	4.29 ± 1.47	12.32 ± 4.37	5.34 ± 1.13
Q2	20.40 ± 6.14	10.60 ± 4.44	8.14 ± 3.31	5.00 ± 1.78	4.31 ± 1.43	12.57 ± 4.07	5.42 ± 1.01
Q3	22.18 ± 5.80	11.18 ± 4.06	9.14 ± 3.09	5.41 ± 1.74	4.67 ± 1.37	13.83 ± 3.60	5.59 ± 0.91
Q4	23.16 ± 5.75	11.82 ± 3.70	9.52 ± 3.15	5.60 ± 1.67	4.74 ± 1.33	14.05 ± 3.54	5.66 ± 0.79
Poultry							
Q1	20.25 ± 6.22	10.57 ± 4.36	7.97 ± 3.37	5.09 ± 1.78	4.32 ± 1.43	12.38 ± 4.28	5.34 ± 1.11
Q2	20.41 ± 6.28	10.55 ± 4.42	8.26 ± 3.28	4.92 ± 1.84	4.30 ± 1.49	12.78 ± 3.96	5.38 ± 1.09
Q3	22.32 ± 6.02	11.30 ± 3.89	9.16 ± 3.28	5.47 ± 1.68	4.66 ± 1.33	13.63 ± 3.72	5.57 ± 0.91
Q4	23.11 ± 6.04	11.84 ± 4.08	9.38 ± 3.29	5.58 ± 1.73	4.72 ± 1.38	13.84 ± 3.95	5.68 ± 0.81
Fish							
Q1	20.13 ± 6.05	10.50 ± 4.30	7.92 ± 3.24	5.07 ± 1.72	4.25 ± 1.40	12.10 ± 4.25	5.34 ± 1.13
Q2	20.64 ± 6.23	10.31 ± 4.49	8.36 ± 3.28	5.14 ± 1.76	4.41 ± 1.46	12.72 ± 4.05	5.45 ± 1.02
Q3	22.71 ± 6.12	11.56 ± 4.16	9.30 ± 3.27	5.56 ± 1.66	4.73 ± 1.37	13.84 ± 3.85	5.59 ± 0.91
Q4	22.61 ± 6.21	11.88 ± 3.74	9.18 ± 3.43	5.29 ± 1.92	4.61 ± 1.40	13.96 ± 3.64	5.58 ± 0.89
Eggs							
Q1	21.53 ± 6.48	11.01 ± 4.30	8.76 ± 3.39 *	5.14 ± 1.93	4.45 ± 1.47	13.14 ± 4.01	5.54 ± 0.96
Q2	21.92 ± 6.02	11.32 ± 4.03	8.85 ± 3.27	5.35 ± 1.76	4.58 ± 1.38	13.56 ± 3.75	5.52 ± 0.94
Q3	21.63 ± 6.18	11.14 ± 4.25	8.68 ± 3.30	5.37 ± 1.72	4.54 ± 1.42	13.08 ± 4.13	5.48 ± 1.04
Q4	21.04 ± 6.33	10.80 ± 4.32	8.49 ± 3.46	5.20 ± 1.69	4.43 ± 1.40	12.84 ± 4.20	5.43 ± 1.03
Dairy							
T0	20.94 ± 6.46	10.96 ± 4.34	8.32 ± 3.45	5.10 ± 1.83	4.39 ± 1.44	12.82 ± 4.16	5.40 ± 1.06
T1	21.15 ± 6.17	10.55 ± 4.27	8.67 ± 3.29	5.18 ± 1.81	4.44 ± 1.44	12.99 ± 3.87	5.50 ± 0.99
T2	22.79 ± 5.98	11.71 ± 3.82	9.29 ± 3.24	5.58 ± 1.69	4.71 ± 1.38	13.89 ± 3.85	5.65 ± 0.90
T3	23.00 ± 5.36	11.39 ± 4.00	9.58 ± 2.90	5.70 ± 1.50	4.78 ± 1.30	13.95 ± 3.63	5.68 ± 0.76
Nuts							
T0	20.69 ± 6.16	10.80 ± 4.30	8.15 ± 3.32	5.14 ± 1.72	4.38 ± 1.41	12.46 ± 4.17	5.39 ± 1.08
T1	20.80 ± 6.36	10.36 ± 4.40	8.53 ± 3.39	5.02 ± 1.96	4.37 ± 1.46	13.09 ± 4.00	5.48 ± 0.99
T2	22.27 ± 6.10	11.43 ± 4.06	9.20 ± 3.29	5.35 ± 1.79	4.58 ± 1.42	13.68 ± 3.61	5.55 ± 0.95
T3	23.44 ± 6.03	12.03 ± 3.83	9.59 ± 3.22	5.72 ± 1.62	4.83 ± 1.35	14.32 ± 3.77	5.68 ± 0.80

^a^: MIS = Memory index score, EIS = Executive index score, VIS = Visuospatial index score, LIS = Language index score, AIS = Attention index score, OIS = Orientation index score. Global cognitive function score and cognitive domain scores are expressed as mean ± SD, evaluated by Montreal Cognitive Assessment (MoCA, Beijing Version). Q1–Q4 are consumption levels of foods (except dairy and nuts) grouped by their quartile consumption; T0 = non-consumer group for dairy and nuts, T1–T3 are consumption levels of dairy and nuts grouped by the tertile consumption of consumers, expressed as mean ± SD. *: *p* value > 0.05, examined by Wilcoxon signed rank test and Kruskal–Wallis H test.

**Table 5:** Associations of food consumption with global cognitive score and cognitive domain subscores using quantile regression model ^a^.

Foods	Global Cognitive Function		Cognition Domain Scores											
			MIS		EIS		VIS		LIS		AIS		OIS	
	*β*	*p* Value	*β*	*p* Value	*β*	*p* Value	*β*	*p* Value	*β*	*p* Value	*β*	*p* Value	*β*	*p* Value
Rice														
Q2	0.79	0.008	0.51	0.016	0.44	0.002	0.08	0.257	0.00	0.952	−0.10	0.609	0.00	0.963
Q3	2.26	<0.001	1.19	<0.001	1.00	<0.001	0.25	<0.001	0.26	<0.001	1.10	<0.001	0.00	0.564
Q4	0.51	0.056	0.21	0.378	0.49	0.003	−0.19	0.028	−0.12	0.116	0.32	0.109	0.00	1.000
Wheat														
Q2	−0.88	0.003	−0.50	0.004	−0.60	<0.001	0.00	0.952	−0.10	0.194	−0.30	0.087	0.00	1.000
Q3	−1.01	<0.001	−0.37	0.013	−0.47	0.002	−0.19	0.021	−0.03	0.626	−0.45	0.007	0.00	1.000
Q4	−1.16	<0.001	−0.19	0.307	−0.64	<0.001	−0.06	0.531	−0.02	0.787	−0.38	0.106	0.00	1.000
Tubers														
Q2	−0.03	0.917	−0.18	0.299	0.06	0.688	0.00	0.974	0.13	0.092	−0.01	0.973	0.00	0.945
Q3	−0.01	0.964	−0.27	0.267	0.00	0.990	0.04	0.616	0.13	0.062	0.02	0.916	0.00	1.000
Q4	−0.11	0.693	−0.24	0.161	0.07	0.642	−0.01	0.898	0.08	0.269	0.14	0.442	0.00	0.979
Legumes														
Q2	0.68	0.018	0.30	0.153	0.39	0.015	0.32	<0.001	0.28	<0.001	0.36	0.055	0.00	1.000
Q3	1.12	<0.001	0.64	0.002	0.35	0.009	0.47	<0.001	0.19	0.013	0.25	0.186	0.00	1.000
Q4	1.57	<0.001	1.04	<0.001	0.61	<0.001	0.60	<0.001	0.29	<0.001	1.09	<0.001	0.00	1.000
Fresh vegetables														
Q2	0.22	0.462	−0.03	0.868	−0.07	0.672	0.06	0.503	−0.09	0.201	−0.04	0.823	0.00	0.988
Q3	0.65	0.032	0.76	<0.001	0.12	0.458	0.03	0.807	−0.04	0.585	0.75	<0.001	0.00	0.926
Q4	1.58	<0.001	1.14	<0.001	0.64	<0.001	0.27	0.010	0.15	0.041	1.16	<0.001	0.00	1.000
Fresh fruit														
Q2	0.00	1.000	−0.20	0.232	−0.01	0.962	0.07	0.280	0.06	0.325	0.11	0.573	0.00	0.981
Q3	1.54	<0.001	0.76	<0.001	0.79	<0.001	0.44	<0.001	0.26	0.001	0.86	<0.001	0.00	1.000
Q4	1.16	<0.001	0.41	0.054	0.56	<0.001	0.41	<0.001	0.15	0.055	0.83	<0.001	0.00	1.000
Pork														
Q2	1.03	<0.001	0.40	0.032	0.30	0.039	−0.01	0.861	0.22	0.001	0.56	0.003	0.00	1.000
Q3	1.70	<0.001	0.83	<0.001	0.69	<0.001	0.19	0.020	0.22	0.002	0.88	<0.001	0.00	0.874
Q4	1.46	<0.001	1.10	<0.001	0.45	0.014	0.02	0.769	0.11	0.116	0.89	<0.001	0.00	1.000
Beef or mutton														
Q2	−0.42	0.169	−0.53	0.069	−0.28	0.150	−0.18	0.066	−0.06	0.437	0.18	0.431	0.00	0.923
Q3	0.42	0.144	−0.14	0.310	0.38	0.004	0.12	0.095	0.20	0.002	0.79	<0.001	0.00	1.000
Q4	0.66	0.016	0.06	0.723	0.20	0.130	0.08	0.260	0.07	0.273	0.42	0.023	0.00	0.969
Poultry														
Q2	0.00	0.993	−0.05	0.780	−0.01	0.939	−0.17	0.021	0.03	0.695	0.42	0.018	0.00	1.000
Q3	0.68	0.010	0.00	1.000	0.26	0.042	0.01	0.875	0.08	0.238	0.38	0.028	0.00	0.994
Q4	1.69	<0.001	0.75	<0.001	0.61	<0.001	0.00	1.000	0.25	<0.001	0.67	<0.001	0.00	1.000
Fish														
Q2	0.09	0.755	−0.08	0.717	0.18	0.286	−0.05	0.507	0.11	0.060	0.69	<0.001	0.00	0.948
Q3	1.51	<0.001	0.74	<0.001	0.64	<0.001	0.26	0.003	0.25	<0.001	1.15	<0.001	0.00	1.000
Q4	1.60	<0.001	0.87	<0.001	0.66	<0.001	0.06	0.545	0.23	0.004	1.33	<0.001	0.00	1.000
Eggs														
Q2	0.01	0.985	0.12	0.493	−0.04	0.775	0.28	<0.001	0.14	0.046	0.34	0.066	0.00	1.000
Q3	0.10	0.682	0.30	0.120	−0.13	0.328	0.21	0.010	0.10	0.110	−0.03	0.846	0.00	1.000
Q4	−0.44	0.127	−0.01	0.957	−0.28	0.088	−0.01	0.877	0.04	0.561	−0.12	0.536	0.00	1.000
Dairy														
T1	−0.86	0.001	−1.00	<0.001	−0.25	0.099	−0.29	<0.001	−0.18	0.020	−0.62	0.001	0.00	1.000
T2	0.26	0.405	−0.08	0.551	0.05	0.757	0.10	0.193	0.10	0.196	−0.10	0.632	0.00	0.998
T3	0.18	0.568	−0.17	0.382	0.22	0.165	0.14	0.068	0.08	0.387	0.10	0.610	0.00	1.000
Nuts														
T1	−0.58	0.048	−0.65	0.003	−0.02	0.899	0.00	1.000	−0.17	0.012	0.41	0.018	0.00	1.000
T2	0.49	0.048	0.25	0.144	0.35	0.008	0.06	0.458	0.02	0.706	0.60	<0.001	0.00	1.000
T3	1.87	<0.001	0.71	<0.001	0.64	<0.001	0.50	<0.001	0.34	<0.001	1.39	<0.001	0.00	1.000

^a^: Q = quartile, reference = Q1; T = tertile, reference = T0 (non-consumer). Adjusted for age, gender, residential area, education level, employment, income level, physical activity, smoking, alcohol intake, sleep duration status, energy, disease history, obesity, and central obesity.

## Data Availability

No additional data are available.

## Supplementary Materials

The following are available online at https://www.mdpi.com/article/10.3390/nu13041341/s1, Table S1: Food grouping used in the dietary consumption analysis, Table S2: Characteristics and prevalence of MCI and its subtypes among Chinese adults aged 55 years and above in four provinces in CCSNSD 2018–2019, Table S3: Differences in global cognitive scores and cognitive domain subscores by characteristics among Chinese adults aged 55 years and above in four provinces in CCSNSD 2018–2019.
