# Supplementary material for: Diet–Cognition Associations Differ in Mild Cognitive Impairment Subtypes
Source: Nutrients. 2021 Apr 17;13(4):1341. doi: 10.3390/nu13041341 (PMC8073801; doi:10.3390/nu13041341)
Supplement: Supplementary file 1 [file nutrients-13-01341-s001.zip › nutrients-1172701-supplementary.pdf]

Table S1. Food grouping used in the dietary consumption analysis

Food group	Detailed food items
Rice	Cooked white rice, cooked rice with assorted mixtures, rice-noodle
Wheat	Non-fried noodles (bread, steamed buns, noodles, dumplings)
Tuber	Sweet potato, potato, taro
Legume	Soybeans, mung beans, red beans, soy milk, soy flour, tofu
Fresh vegetable	Fresh legume vegetables, tomatoes, peppers, melon vegetables, green leafy vegetables, cabbage and other leafy vegetables, cruciferous vegetables
Fresh fruit	Orange fruits, melon fruits, berry fruits, all other fresh fruits
Pork	Lean pork, fatty pork
Beef or mutton	Beef, lamb, mutton
Poultry	Chicken, duck, goose, pigeon, quail
Fish	Sea fish, freshwater fish
Egg	Fresh egg, salted egg, preserved egg
Dairy	Liquid milk, milk powder, yogurt, cheese
Nut	Peanuts, melon seeds, pumpkin seeds, watermelon seeds

Table S2. Characteristics and prevalence of MCI and its subtypes among Chinese adults aged 55 years and above in four provinces in CCSNSD 2018-2019[a]

Characteristics	N (%)	MCI[b]		*P* value	MCI subtypes[b]				*P* value
		Yes	No		aMCI-SD	naMCI-SD	aMCI-MD	naMCI-MD	
Total	4309(100.0)	42.6	57.4		4.8	6.9	8.2	6.0	
Age group (years)				<0.001					<0.001
55-64	1586(36.8)	38.1	61.9		4.9	6.6	5.2	3.7	
65-74	1864(43.3)	42.0	58.0		4.8	7.1	8.3	5.7	
≥75	859(19.9)	51.9	48.1		4.4	7.1	13.7	11.1	
Gender				0.476					<0.001
male	1956(45.4)	42.0	58.0		6.0	7.4	6.6	4.1	
female	2353(54.6)	43.1	56.9		3.7	6.5	9.6	7.6	
Resident area				<0.001					<0.001
urban	2130(49.4)	38.5	61.5		4.6	6.2	5.0	5.2	
rural	2179(50.6)	46.5	53.5		4.9	7.5	11.4	6.8	
Education level				<0.001					<0.001
illiteracy	613(14.2)	39.0	61.0		0.2	1.0	23.3	14.5	
≤Primary school	1784(41.4)	38.6	61.4		4.7	8.5	9.6	7.1	
≥Secondary school	1912(44.4)	47.4	52.6		6.3	7.3	2.1	2.2	
Current employment				0.011					0.165
yes	772(17.9)	38.5	61.5		5.7	5.6	7.3	5.1	
no	3537(82.1)	43.5	56.5		4.6	7.2	8.5	6.2	
Monthly household income per capital (RMB)				<0.001					0.005
<1000	1017(23.6)	54.2	45.8		5.5	10.1	14.4	9.9	
1000-3999	2653(61.6)	42.2	57.8		4.8	6.5	7.4	5.4	
≥4000	639(14.8)	25.5	74.5		3.4	3.4	2.2	2.3	
Physical activity level				<0.001					<0.001
low	1431(33.2)	38.1	61.9		3.0	7.8	7.5	5.7	
medium	1442(33.5)	43.7	56.3		4.8	5.9	9.6	6.5	
high	1436(33.3)	45.9	54.1		6.5	7.0	7.5	5.8	
Smoking				0.006					<0.001

ever/current	1036(24.0)	46.2	53.8		7.1	8.8	8.0	4.5	
never	3273(76.0)	41.4	58.6		4.0	6.3	8.3	6.5	
Alcohol intake				0.214					<0.001
ever/current	743(17.2)	40.5	59.5		7.0	7.8	6.2	2.8	
never	3566(82.8)	43.0	57.0		4.3	6.7	8.7	6.7	
Meeting sleep duration recommendation				0.187					<0.001
yes	1505(34.9)	43.9	56.1		6.5	6.9	10.9	5.4	
no	2804(65.1)	41.8	58.2		3.8	6.9	6.8	6.3	
Medical history				0.004					0.064
yes	1562(36.3)	45.5	54.5		5.2	6.5	9.9	7.2	
no	2747(63.8)	40.9	59.1		4.5	7.1	7.3	5.4	
Obesity				0.165					0.840
yes	568(13.2)	45.2	54.8		4.8	6.7	9.2	6.9	
no	3741(86.8)	42.2	57.8		4.8	6.9	8.1	5.9	
central obesity				0.969					0.822
yes	2005(46.5)	42.6	57.4		4.9	6.8	7.8	5.8	
no	2304(53.5)	42.5	57.5		4.6	6.9	8.6	6.2	
Energy (kcal)[c]	1522(620.7)	1484	1550	0.003	1590	1489	1464	1392	0.016

[a]: CCSNSD= Community-based Cohort Study on Nervous System Diseases, aMCI-SD=amnestic MCI single domain，naMCI-SD=nonamnestic MCI single domain，aMCI-MD=amnestic MCI multiple domains，naMCI-MD=nonamnestic MCI multiple domains. Chi-Square Test was applied for the analysis of categorical variables, Wilcoxon Signed Rank Test or Kruskal-Wallis H Test were employed for the analysis of continuous variables. *P* value < 0.05 was considered to be statistically significant.

[b]: expressed as % (prevalence).

[c]: expressed as mean.

Table S3. Differences in global cognitive score and cognitive domain subscores by characteristics among Chinese adults aged 55 years and above in four provinces in CCSNSD 2018-2019[a]

Characteristics	Global cognitive function	Cognition domain scores					
		MIS	EIS	VIS	LIS	AIS	OIS
Total	21.53±6.26	11.07±4.23	8.69±3.36	5.26±1.78	4.50±1.42	13.16±4.03	5.49±0.99
Age group (years)							
55-64	22.85±5.65	11.70±3.79	9.37±3.06	5.63±1.56	4.74±1.28	13.93±3.71	5.65±0.83
65-74	21.52±6.11	11.04±4.22	8.72±3.29	5.24±1.76	4.50±1.40	13.20±3.90	5.50±0.96
≥75	19.11±6.90	9.97±4.76	7.39±3.63	4.63±2.00	4.07±1.59	11.66±4.42	5.19±1.24
P value	<0.001	<0.001	<0.001	<0.001	<0.001	<0.001	<0.001
Gender							
male	22.16±5.68	11.16±4.09	9.13±3.06	5.48±1.62	4.60±1.34	13.48±3.84	5.58±0.87
female	21.00±6.66	10.99±4.34	8.33±3.54	5.08±1.88	4.42±1.48	12.89±4.16	5.42±1.08
P value	<0.001	0.790	<0.001	0.001	<0.001	<0.001	<0.001
Resident area							
urban	22.84±5.84	11.66±3.86	9.36±3.17	5.54±1.74	4.73±1.33	13.93±3.72	5.65±0.84
rural	20.25±6.39	10.49±4.48	8.05±3.40	5.00±1.78	4.28±1.47	12.41±4.17	5.34±1.10
P value	<0.001	<0.001	<0.001	<0.001	<0.001	<0.001	<0.001
Education level							
illiteracy	15.71±6.81	8.53±5.20	5.49±3.38	3.80±2.01	3.44±1.53	9.58±4.57	4.80±1.47
≤Primary school	20.81±5.95	10.84±4.36	8.26±3.19	4.99±1.75	4.37±1.38	12.95±3.83	5.45±0.98
≥Secondary school	24.07±4.76	12.09±3.27	10.13±2.59	5.99±1.30	4.96±1.20	14.50±3.20	5.75±0.65
P value	<0.001	<0.001	<0.001	<0.001	<0.001	<0.001	<0.001
Current employment							
yes	22.41±5.95	11.42±4.18	9.12±3.27	5.27±1.88	4.74±1.38	13.98±3.59	5.59±0.86
no	21.34±6.31	10.99±4.24	8.60±3.37	5.26±1.76	4.45±1.42	12.98±4.09	5.47±1.02
P value	<0.001	0.002	<0.001	0.390	<0.001	<0.001	0.011
Monthly household income per capital (RMB)							
<1000	18.16±6.17	9.60±4.72	6.92±3.18	4.45±1.92	3.93±1.51	11.38±4.23	5.08±1.29
1000-3999	22.05±5.97	11.29±4.06	8.95±3.24	5.41±1.67	4.60±1.36	13.43±3.88	5.59±0.88
≥4000	24.75±5.09	12.48±3.33	10.46±2.82	5.95±1.49	4.99±1.19	14.84±3.20	5.74±0.66
P value	<0.001	<0.001	<0.001	<0.001	<0.001	<0.001	<0.001
Physical activity level							
low	21.74±6.41	11.33±4.28	8.72±3.38	5.35±1.70	4.42±1.43	13.01±4.30	5.48±1.02
medium	21.31±6.38	10.86±4.28	8.63±3.47	5.23±1.83	4.48±1.44	12.96±4.05	5.48±1.02
high	21.54±5.98	11.02±4.11	8.74±3.21	5.21±1.79	4.60±1.39	13.51±3.69	5.53±0.94
P value	0.162	<0.001	0.906	0.116	0.001	0.005	0.682
Smoking							
ever/current	21.50±5.65	10.64±4.30	8.87±3.03	5.37±1.66	4.51±1.36	13.17±3.81	5.52±0.91
never	21.54±6.44	11.21±4.20	8.64±3.45	5.23±1.81	4.50±1.44	13.15±4.09	5.49±1.02
P value	0.188	<0.001	0.358	0.188	0.787	0.510	0.558
Alcohol intake							

ever/current	22.59±5.32	11.13±4.05	9.47±2.83	5.61±1.57	4.72±1.31	13.87±3.54	5.61±0.81
never	21.31±6.42	11.06±4.27	8.53±3.43	5.19±1.81	4.45±1.44	13.01±4.11	5.47±1.03
P value	<0.001	0.777	<0.001	<0.001	<0.001	<0.001	0.008
Meeting sleep duration recommendation							
yes	21.81±6.08	11.28±4.01	8.84±3.28	5.32±1.74	4.53±1.40	13.26±3.93	5.54±0.95
no	21.01±6.56	10.68±4.59	8.42±3.48	5.17±1.84	4.44±1.46	12.98±4.20	5.40±1.06
P value	<0.001	<0.001	<0.001	0.025	0.127	0.145	<0.001
Disease history							
yes	20.69±6.48	10.61±4.40	8.30±3.48	5.10±1.84	4.37±1.48	12.62±4.24	5.41±1.07
no	22.01±6.08	11.33±4.11	8.92±3.26	5.36±1.73	4.57±1.38	13.47±3.87	5.54±0.94
P value	<0.001	<0.001	<0.001	<0.001	<0.001	<0.001	<0.001
Obesity							
yes	20.89±6.34	10.69±4.38	8.41±3.43	5.24±1.67	4.45±1.35	12.68±4.22	5.43±1.05
no	21.63±6.24	11.13±4.20	8.74±3.34	5.27±1.79	4.51±1.43	13.23±3.99	5.50±0.99
P value	0.006	0.018	0.036	0.240	0.108	0.004	0.077
central obesity							
yes	21.54±6.28	11.07±4.22	8.68±3.39	5.30±1.74	4.54±1.40	13.13±4.11	5.50±1.00
no	21.52±6.24	11.07±4.23	8.70±3.33	5.23±1.81	4.46±1.43	13.18±3.95	5.49±0.99
P value	0.852	0.956	0.962	0.341	0.074	0.947	0.684

[a]: CCSNSD= Community-based Cohort Study on Nervous System Diseases, MIS=Memory index score，EIS=Executive index score，VIS=Visuospatial index score，LIS=Language index score，AIS=Attention index score，OIS=Orientation index score. Scores of global cognitive function and domains were expressed as mean±SD, evaluated by Montreal Cognitive Assessment (MoCA, Beijing Version). *P* value < 0.05 was considered to be statistically significant, examined by Wilcoxon Signed Rank Test or Kruskal-Wallis H Test.
